# Potential impact of eradicating malaria on gender inequality within agricultural households in sub-Saharan Africa

**DOI:** 10.12688/gatesopenres.13154.1

**Published:** 2020-07-22

**Authors:** Derek W. Willis, Nick Hamon

**Affiliations:** 1 Center for Research On Environmental Decisions, Columbia University, New York, NY, 10027, USA; 2 Global Health, OnFrontiers, New York, NY, 10010, USA; 3 Innovative Vector Control Consortium, Liverpool, L3 5QA, UK

**Keywords:** malaria, gender inequality, Africa, agricultural households, time poverty

## Abstract

The international development community has shown an increased interest in the links between malaria and gender inequality over the past two decades. Working towards the ambitious goal of eradicating malaria by 2040, suppressing the malaria burden could accelerate progress in reducing gender inequality within agricultural households in sub-Saharan Africa. Although numerous studies have examined narrow aspects of the relationship between malaria and gender inequality, little progress has been made in understanding how eliminating malaria could affect gender inequality within agricultural households. This Open Letter focuses on the amount of time women farmers dedicate to caregiving for malaria cases among children in agricultural households, and how reducing time spent on this activity could reduce gender inequalities and impact agricultural productivity. We argue that a research agenda is needed to inform a multi-disciplinary approach to gain this understanding. We conclude by discussing the means through which a reduction in gender inequalities in agricultural households could impact the effectiveness of vector control interventions.

## Disclaimer

The views expressed in this article are those of the author(s). Publication in Gates Open Research does not imply endorsement by the Gates Foundation.

## Introduction

In 2007, Bill Gates asked leading global health organizations to embrace the ambitious goal of eradicating malaria ^1^. To achieve this goal over the next two to three decades, funding for malaria eradication initiatives is critical. Unfortunately, during and after the first Global Malaria Eradication Campaign (1955 to 1969) there was a resurgence of malaria, primarily due to insufficient funding ^2^. Generating and maintaining political and financial support from donors will likely play a key role in the long-term success of the current eradication initiative ^3, 4^. Understanding the potential economic and social impact of eradicating malaria on households could affect funding decisions by international donors.

The economic and social impact of malaria on agricultural households has been studied extensively. The vast majority of these studies have quantified the impact of malaria on the agricultural productivity of male farmers (or male-headed agricultural households) or the impact of malaria on caregiving time by women in these households.

Following the discovery of the malaria transmission cycle in 1897, Van Dine ^5^ undertook one of the first studies to examine the economic impact of malaria at the agricultural household level. By examining data collected from farmers in Madison Parish, Louisiana, he quantified the number of workdays lost by individuals in a household due directly to malaria.

More recent studies have quantified the impact of malaria on the harvest values of farmers ^6, 7^. The first of these two studies ^6, 7^ examined a small number of cabbage farmers in Côte d'Ivoire and found a 53% reduction in revenue among farmers who missed more than two days of work due to malaria when compared to farmers who missed fewer than two days of work. The second and more recent of these two studies examined farmers in Zambia and found that the provision of long-lasting insecticidal nets (LLINs) to agricultural households increased harvest value by 14.7% ^7^. Although the specific causal pathway through which LLINs increased harvest value could not be precisely identified, the study suggests that a reduction in work days lost due to malaria morbidity is the most plausible explanation ^7^. Both studies were conducted in less than one year. The final analysis of Côte d'Ivoire data included just 12 farmers ^6^, while the Zambia data included a study population in which more than 75% of farmers were male ^7^. To date, no studies have used longitudinal data over multiple years to analyze potential differences in the impact of malaria on the productivity of male and female farmers within a household.

In the 1990s and 2000s, several studies identified women as the primary caregivers for malaria cases in sub-Saharan Africa ^8– 19^. Extensive literature has identified gender inequalities in parents’ decisions regarding how resources are allocated to children’s health and education; women are more likely than men to devote resources to improving the health and education of children within a household ^20– 30^.

In a recent survey of Kenyan women, Ernst *et al*. ^31^ found that women have a higher awareness of malaria vectors and disease transmission. The authors argued that women should play a more prominent role in vector control decisions due to their interest in family wellbeing. They further argued that women are better able to integrate appropriate vector control interventions into the activities of the household ^31^.

While the relationship between malaria and gender in Africa has been studied extensively (a search of the electronic database, PubMed, using the keywords ‘malaria’, ‘gender’, and ‘Africa’ identified 1,100 journal articles), none of these studies have examined the potential impact of eliminating malaria on gender inequalities in agricultural productivity. This is surprising given the approximately 54 million agricultural households in malarious regions of sub-Saharan Africa ^32^ and interest by the international development community in gender inequality ^33– 35^. Most malaria cases in sub-Saharan Africa occur in children ^36, 37^; and women farmers devote more time to caregiving for malaria cases among children than men ^9, 11, 16, 19^. Given the findings from Côte d'Ivoire ^6^ and Zambia ^7^, malaria may have a disproportionate impact on the productivity of female farmers than male farmers. Reducing the amount of caregiving time necessary for malaria cases could increase the agricultural productivity of women farmers in agricultural households.

Over the last two decades, calls for research agendas focusing on malaria eradication have proven to be effective in stimulating research. In 2011, for example, the malERA Consultative Group on Drugs issued a call to identify and address new research questions related to antimalarial drugs that would not have been prioritized without the goal of eradication ^38^. This agenda facilitated clinical development of new classes of antimalarial compounds ^39– 41^, as well as the development and dissemination of guidelines for implementing mass drug administration ^39, 42^. We believe that a similar research initiative is necessary to examine how eradicating malaria could affect the welfare of agricultural households in sub-Saharan Africa. In this Open Letter, we highlight the importance of an agenda linking two fields of research: (1) the impact of time poverty on the productivity of women farmers, and (2) gender inequalities in caregiving time for malaria cases within a household. We conclude by discussing the potential impact of reducing gender inequality in agricultural households on the effectiveness of vector control interventions and present a set of recommendations for future work.

## Gender inequality topics to prioritize for conceptual framework

In this section, we first describe research on gender inequalities in agricultural productivity and the challenges that time poverty imposes on the productivity of women farmers. Next, we describe research on gender inequalities in caregiving time for malaria cases. We then use a hypothetical household with three children to estimate the total time that a woman might devote to malaria-associated childcare during the period of time when her children are aged 15 years old and under.

In previous work, we estimated that there are approximately 324 million individuals living in agricultural households in malarious regions of sub-Saharan Africa ^43^. We define an agricultural household as a household with less than 10 hectares of farming area. This is the same definition used in our previous work ^32, 44, 45^. and is consistent with the definition of agricultural households used in a 2010 agricultural census conducted in Ethiopia:

A household is considered an agricultural household when at least one member of the household is engaged in growing crops and/or raising livestock in private or in combination with others ^46^.

Consistent with how these terms have been used in other studies ^47^, we use “female farmers” and “women farmers” interchangeably to identify women who are responsible for making important decisions for a specific agricultural plot.

### Impact of time poverty on productivity of women farmers

Peterman *et al.* reviewed the literature on gender inequalities in agricultural productivity for sub-Saharan Africa, finding a 25% difference in the productivity of female and male farmers ^48^. A separate report focused on six countries comprising more than 40% of the population in sub-Saharan Africa and found that, on average, the difference in agricultural productivity between women and men ranged from 66% in Niger to 23% in Tanzania, accounting for differences in geographic factors and plot size ^47^.

***Agricultural inputs.*** Research shows that women farmers in sub-Saharan Africa often experience greater challenges in gaining access to agricultural inputs (e.g., land, labor, fertilizer, seeds, information) compared to men, leading to lower levels of productivity ^49– 69^. Women are often required to farm land that they do not own, such as land belonging to their husband or male relatives ^51^. They face disincentives to invest in improving this land, which can affect the value of their harvests ^51, 70^. However, the threat of fallow land being appropriated by the community incentivizes women to continually cultivate the land, which leads to a deterioration in soil fertility ^71^. Women farmers also often experience challenges in accessing information regarding methods to increase their productivity, due to a lack of access to agricultural extension workers or illiteracy. A study in Burkina Faso found that access to female agricultural extension workers was critical for increasing the productivity of women farmers ^72^.

In 2011, the Food and Agriculture Organization published an influential report titled *The State of Food and Agriculture 2010–2011: Women in Agriculture: Closing the Gender Gap for Development,* which highlighted the importance of addressing gender inequities to increase the productivity of women farmers. To create incentives for women to make investments that increase agricultural productivity, protective policies for women’s land rights are needed ^73^. In Ethiopia, the introduction of joint land registration gave women the formal right to the land that they farm ^74, 75^. This policy led to increased investments in the productivity of land farmed by women ^74^. Organizations are developing initiatives that focus on providing credit and agricultural inputs to women; self-help groups developed by women are increasing access to financial services and technologies to help increase productivity ^76^.

***Time poverty.*** Increasing the access and use of agricultural inputs by women farmers is not, in and of itself, sufficient to increase agricultural productivity. A common expectation in agricultural households is that women will devote more time for caregiving than men ^77^. This is problematic given that an extensive body of research has found that the amount of time that women devote to childcare and other household responsibilities negatively impacts their agricultural productivity ^54^. Expectations that women are responsible for household activities is an important example of the broader norms that prevent women from achieving the levels of agricultural productivity reached by men when provided access to similar levels of agricultural inputs ^54, 78– 83^. Gyasi refers to women’s diverse household activities as a “zero sum game” in which the more time women devote to a new activity within the household, the less time they have available for other commitments ^77, 84, 85^.

de Schutter notes that women can become trapped in a “care economy” that leads to a vicious cycle in which time poverty prevents women from achieving economic independence:

Women are less economically independent, are exposed to violence and have a weaker bargaining position within the household and the community. As a result, they continue to assume a highly unequal share of tasks and family responsibilities within the household - taking care of the children and the elderly or the sick, fetching wood and water, buying and preparing the food. This “care economy” for which they remain chiefly responsible results in time poverty for women ^84^.

Although the issue of time poverty among women in sub-Saharan Africa is clear, the policy options to address the situation are limited. A 2006 World Bank report highlighted the need for investments in infrastructure targeted at reducing the time necessary for household tasks rather than infrastructure focused on income-generating activities:

It is critical to focus attention on development outcomes (informing the “results agenda”) that time poverty most affects. This in turn requires much more focus on technology, including labor-saving technology accessible to women to reduce the burden and drudgery of household tasks. In this context, the renewed focus on infrastructure, for example in the World Bank’s Africa Action Plan, while welcome, needs to be directed toward meeting the speciﬁc needs of the house-hold economy ^79^.

Bold statements were also made in 2010 by high-level government officials regarding the need for labor-saving technologies and infrastructure in rural areas to reduce the amount of time women devote to domestic activities ^84, 86^, which would, in turn, enable women to experience the benefits of the economic opportunities that are created by agricultural growth ^73^. A 2014 World Bank report recommended measures to reduce the gender gap between women and men ^47^. de Schutter states that:

In both rural and urban areas, measures would include the establishment or strengthening of child-care services and care for the elderly or persons with illness/disability ^84^

A 2015 study in the Western Democratic Republic of Congo (DRC) used a survey of 2,931 agricultural households to examine how the amount of time women farmers devote to household activities affects their agricultural productivity. On average, the productivity of women farmers was 26% less than men. The study found that women who manage agricultural plots spend 1 hour and 52 minutes more on household activities than male plot managers each day ^87^. In addition, women must devote more time to childcare while they are farming than men, and while the presence of young children in a household does not affect the productivity of men, young children are associated with lower productivity among women farmers. These gender inequalities in agricultural productivity may have a significant impact on the agricultural sector in the DRC, given that more than 70% of economically active women in the DRC work in agriculture ^52^. The Africa Gender Innovation Lab is piloting alternative means of providing childcare services in the Kongo Central region and examining whether the provision of these services affects the productivity of women farmers ^87^.

Household activities can be divided into two components—non-childcare-related activities and childcare-related activities. In the next section, we focus on childcare-related household activities (specifically, illness care for children) and highlight the need to examine how reducing the time women devote to providing care for sick children (especially children sick with malaria) could reduce gender inequalities in agricultural productivity.

### Gender inequalities in caregiving time for malaria cases

Women provide the majority of care for household malaria cases; a study in Ghana found that women provide care in 83% of malaria cases ^88^. The time that women in agricultural households devote to malaria-associated childcare could be an important factor contributing to lower productivity among female farmers. In this section, we provide a brief review of the most relevant literature examining the amount of time women dedicate to malaria-associated caregiving. We also use a model to quantitatively estimate the total number of days a woman might devote to caregiving for malaria cases in children.

Approximately 93% of malaria cases throughout the world occur in sub-Saharan Africa ^37^. The intensity of malaria transmission in rural Africa is often significantly higher than in urban and peri-urban areas.

Within agricultural households in rural sub-Saharan Africa, approximately 50% to 75% of all malaria cases are in children under the age of 16, with morbidity rates among the highest in the world ^36^. A study of the age distribution of cases of *Plasmodium falciparum* malaria in sub-Saharan Africa found that approximately 48% of cases were among children under the age of five years; however, there was wide variation across communities based on malaria transmission intensity ^36^. They estimated less variability in children of school age—somewhere between 20 and 40% of cases ^36^.

A large number of studies in the 1990s and 2000s examined the number of caregiving days provided for each malaria case within agricultural households ^8– 18, 89– 99^. Most estimates for caregiving days necessary per malaria case for households in sub-Saharan Africa ranged from one to six days.

The number of caregiving days provided for each malaria case often depends on the time of year, as the opportunity cost of time will be higher when labor is most needed for agricultural activities. A study in Burkina Faso found that the number of caregiving days provided per malaria case was lower during the rainy season than during the dry season, potentially due to the higher opportunity cost of lost work days ^18^. The requirement of women to provide caregiving would have a greater impact on income during times when labor is greatly needed for cultivating crops ^44^.

***Quantifying inequalities in caregiving days provided by women and men.*** Although numerous studies have attempted to estimate the number of caregiving days required per malaria case in children, no study has estimated the total number of caregiving days provided by adults for all children throughout their childhood. To develop these estimates, we consider a hypothetical agricultural household in an area of intense malaria transmission with two adults and three children. This estimate of five people per household is consistent with average household sizes across much of Africa ^100^.

We defined children as individuals under the age of 16. Children can be divided into two groups—those aged five and under (young children) and those aged six to 15 (older children). Among the 324 million individuals living in agricultural households in malarious regions of sub-Saharan Africa, approximately 52.7 million are under five years of age ^45^.

To illustrate the potential impact of malaria elimination on women’s caregiving responsibilities, we estimate the total number of caregiving days needed per household for malaria cases among children aged 15 and under. We explore nine scenarios for this hypothetical agricultural household. Each scenario includes four parameter values: annual number of malaria cases for young children, caregiving days per case in young children, annual number of malaria cases for older children, and caregiving days per case for older children.

Given that we are estimating the number of malaria cases experienced by children in an agricultural household in an area of intense malaria transmission, we assume that the annual number of malaria cases for young children ranges from one to two. For older children, we assume that the annual number of malaria cases ranges from 0.5 to 1.5. Based on these assumptions, and assuming three young children in a household during one year, the maximum number of cases that that household would experience in that year is six.

While an estimated six malaria cases per household per year may appear high based on national malaria case data, it is consistent with sub-national studies conducted in rural communities. A survey of households in Kasangulu, a rural town in Kongo Central province in the DRC, found that 31.7% of households reported six or more malaria cases over a 12-month period ^101^. In 2017, the DRC (a country with an estimated total population of 84 million that year), experienced an estimated 25 million malaria cases ^102^. However, these estimates are based on passive case detection and likely underestimate the actual number of malaria cases experienced in the country. A 2014 study found that only 34% of the total number of malaria cases in sub-Saharan Africa identified with active case detection would have been recorded with passive case detection ^36^.

We assume that the number of caregiving days provided by adults ranged from three to five for young children, and from two to four for older children. These ranges are consistent with reviews of the relevant literature ^44^. Further, we estimate that women provide care in 80% of cases involving younger children and 70% of cases involving older children. These assumptions are consistent with the finding that women provided care for 83% of malaria cases in Ghana ^88^ and findings from the World Development Report on Gender Equality and Development ^103^.

**Figure 1.**
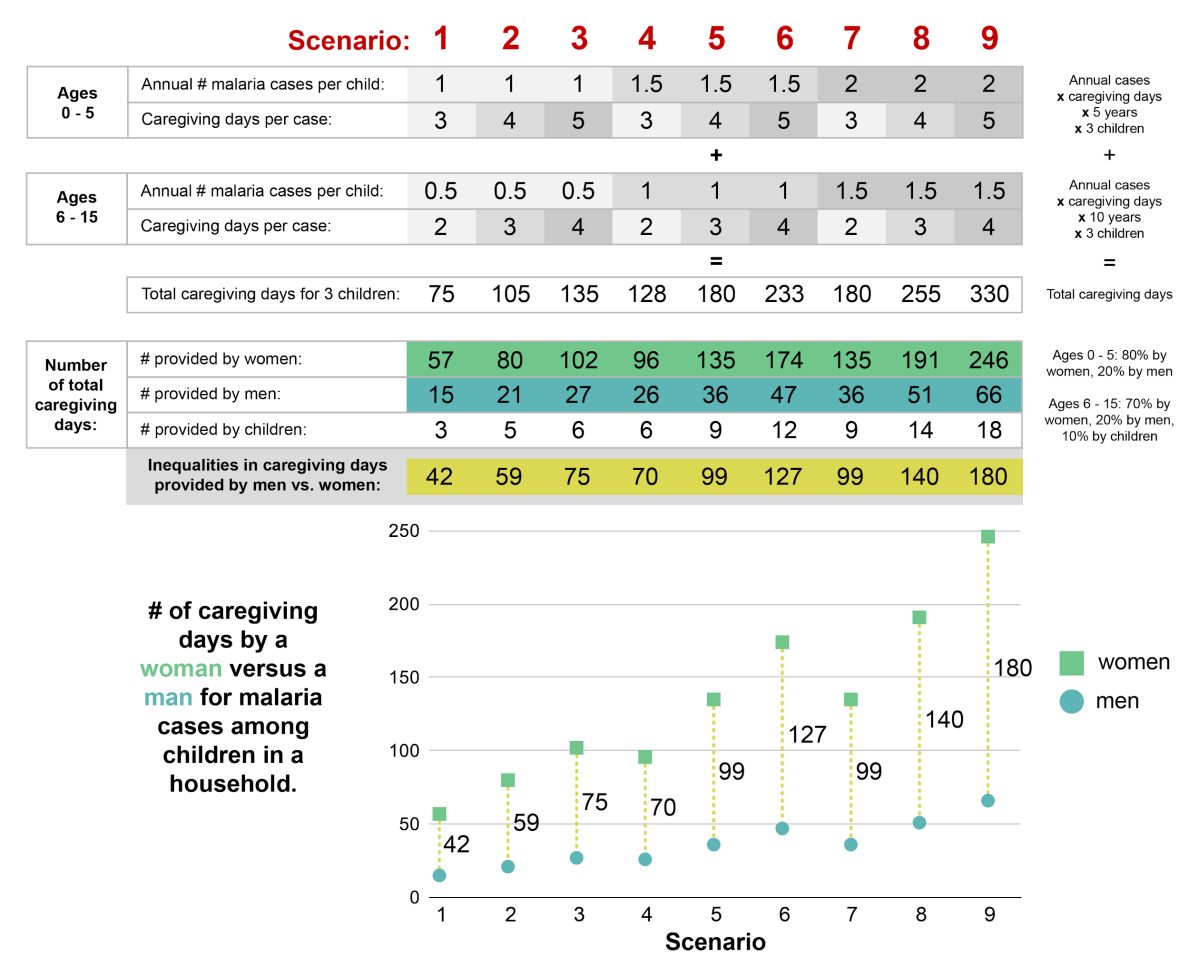
Inequalities in caregiving days provided by women versus men for malaria cases experienced by an agricultural household.. Software used for figure constructions: Adobe Illustrator CC 2020.

Figure 1 summarizes the parameter values and the resulting estimates of gender inequalities in caregiving days for the nine scenarios. Each set of three scenarios (i.e., 1 to 3, 4 to 6, and 7 to 9) have the same parameter values for the number of malaria cases per child for the two age groups but different parameter values for the number of caregiving days per case.

Based on the parameter values we used to develop our estimates, adults in an agricultural household devote a total of approximately 2.5 to 11 months to providing care for malaria cases for three children throughout their childhood (age 15 and under). The inequalities in caregiving time between women and men ranged from approximately 1.5 months to 6 months.

The estimates above suggest that eliminating malaria would significantly reduce the amount of time women must devote to childcare and homemaking; but, how does this compare to previously proposed policies aimed at reducing this time burden among women? Barwell ^104^ used survey data from five villages in sub-Saharan Africa to track the time women spent walking to retrieve water and wood, both essential resources required daily. They estimated the potential time savings for women by improving access to these resources. Improved access meant that a potable water source was accessible within a six-minute walk, consistent with Tanzania’s policy at the time that households should be within 40 meters of a water source. By comparing the time women actually devoted to retrieving water with the time they would spend on this activity if a water source were within a six-minute (i.e., 400 meters) walk, the study quantified the potential time savings of improved water access. For the rural village in Kaya, Burkina Faso, such an innovation would reduce water-retrieving time among women by 125 hours each year. For wood, Barwell considered the potential impact of woodlot creation within a 30-minute walk of households. He found that this policy would reduce the annual time required for wood retrieval by 119 hours per year in that same village.

How do these estimated time savings of 125 hours for water and 119 hours for wood compare to the potential reduction in caregiving time for malaria cases associated with malaria elimination? Assuming that the intensity of malaria transmission in Kaya, Burkina Faso corresponds to the malaria burden depicted in scenario five in Figure 1, eliminating malaria would reduce the number of days women provide malaria-associated childcare (assuming three children) by 135 days throughout the 15 years of childhood. Assigning scenario five for Kaya is a conservative assumption given that the malaria burden in young children in West Africa is generally higher than the burden in East and Southern Africa ^36^. A survey of Kaya in 2010 found that the size of the average household was 6.5 and that malaria was the leading cause of death ^105^. If we assume that each caregiving day represents eight hours of malaria-associated childcare, a conservative assumption, then a woman devotes a total of 1,080 hours providing malaria-associated childcare for three children throughout their childhood. If we assume that all three of these children progress from birth to age 15 over a 20-year period, the average annual reduction in time devoted to malaria-associated caregiving over that period is 54 hours per year. This estimate of 54 hours per year, over a 20-year period, represents approximately 43% (54 hours is 43% of 125 hours) of their time, which could be saved by improving water access in a village like Kaya. Similarly, the estimate represents 45% (54 hours is 45% of 119 hours) of the total time that would be saved by improving access to wood in Kaya.

## A research agenda

The objective of this Open Letter is to highlight the need for a multi-disciplinary research agenda to examine the potential impact of eliminating malaria on gender inequality in agricultural households in sub-Saharan Africa. We have briefly described research in two fields (gender inequalities in agricultural productivity and in caregiving time for malaria cases) that suggests that reducing the time women devote to caregiving for malaria cases among children could increase their agricultural productivity.

Suppressing malaria in rural sub-Saharan Africa may not, in the short term, reduce fundamental gender inequalities related to childcare; there remains a high probability that a sick child will be cared for by a woman rather than a man. However, suppressing and eventually eliminating malaria would provide women more time to focus on increasing their income, and thus, their autonomy. Greater autonomy could, in turn, strengthen women’s ability to make important household decisions (such as those related to vector control strategies and the health and education of children), thereby reducing the negative impact of gender inequality on the welfare of the household.

We conclude by identifying three additional research questions that should be prioritized for the research agenda:

- How would a reduction in caregiving time for malaria cases in agricultural households affect the non-agricultural income-generating activities of women?
- How would an increase in income for women (from agricultural or non-agricultural activities) affect their autonomy to make decisions within the household?
- How would an increase in the autonomy of women to make household vector control decisions impact the effectiveness of vector control interventions?

Given the goal of malaria eradication, new research questions focusing on the potential impact of eradicating malaria on gender inequality within agricultural households in sub-Saharan Africa should be prioritized. Addressing these new research questions will require a shift away from the methodologies that were used in the 1990s when the goal was *control*, rather than eradication, of malaria. Without a research agenda, and the necessary resources to carry out the agenda, the current malaria eradication initiative may fail to recognize how progress towards achieving this ambitious goal is affecting gender inequality in agricultural households.

## Data availability

All data underlying the results are available as part of the article and no additional source data are required.
