# Supplementary material for: Potential impact of eradicating malaria on gender inequality within agricultural households in sub-Saharan Africa
Source: Gates Open Res. 2020 Jul 22;4:114. [Version 1] doi: 10.12688/gatesopenres.13154.1 (PMC7667246; doi:10.12688/gatesopenres.13154.1)
Supplement: Peer review history

# Peer review: Reviewer response for version 1

**DOI:** 10.21956/gatesopenres.14348.r29510

**Published:** 2020-11-13

**Authors:** Itishree Pattnaik

**Affiliations:** 1 Gujarat Institute of Development Research (GIDR), Ahmedabad, Gujarat, India

The paper highlights two important issues; time poverty of women farmers and gender inequality in caregiving for malaria which adversely affects the agricultural productivity of the women farmers in Africa. The issue of time poverty is a larger socio-cultural-economic issue in Africa and Asia. The paper aims to targets these important issues. There are few missing links which the paper needs to focus on.

- The authors establish how caregiving for malaria (time poverty) adversely affects agricultural productivity, which further affects women’s autonomy in household decision making. However, women’s time poverty is a cause of many reproductive and productive activities. It is essential to address all those activities where women spend most of the time. Malaria is not the only disease that adversely affects the agricultural productivity of the farm cultivated by women; there are several other factors that need to be highlighted.
- Women's work on farms might vary seasonally. Women face greater challenges during the peak season or harvesting season compared to the lean season of cropping.
- Apart from malaria, there are several other infectious diseases that prevail in Africa. It is essential to address the time devoted by women for all other diseases. There are also many other seasonal diseases that might affect women’s time more.       
- The literature review is not satisfactory.
- The definitions are not clear such as farm households, women farmers, etc.  

Does the article adequately reference differing views and opinions?

Partly

Are all factual statements correct, and are statements and arguments made adequately supported by citations?

Partly

Is the Open Letter written in accessible language?

Yes

Where applicable, are recommendations and next steps explained clearly for others to follow?

Yes

Is the rationale for the Open Letter provided in sufficient detail?

Yes

Reviewer Expertise:

My areas of research focus on - agricultural sustainable development, gender studies, and migration.

I confirm that I have read this submission and believe that I have an appropriate level of expertise to confirm that it is of an acceptable scientific standard.

# Peer review: Reviewer response for version 1

**DOI:** 10.21956/gatesopenres.14348.r29798

**Published:** 2020-10-29

**Authors:** Manju Rahi

**Affiliations:** 1 Indian Council of Medical Research, New Delhi, India

- Essentially the paper is about how gender differences in context of time spent in care of a malaria affected child adversely affects agricultural productivity in Africa. Gender gap in child rearing and care is a much larger social issue in Africa as in South East Asia and any change would be a gradual process. taking care of a sick child becomes especially demanding on the family more so for a mother, taking away some time from her other activities.
- In Africa, there is a high burden of other infectious diseases as well. If the authors could gather that information and targeting malaria alone would make a substantial difference, this could have been documented by the authors.  
- The authors mentioned that number of days of care provided depended on time of year "opportunity cost of time when labor is needed the most", lesser in rainy season than dry season
- Could the authors comment on impact of lesser time spent on child care on morbidity and mortality of malaria stricken children? Both scenarios needed to be explained. If any adverse impact or no impact. 
- Coming to policies and infrastructure to be women friendly and encouraging economic independence of women, giving examples of provision of potable water within 6 min walk or 400 m. What about malaria? Here some examples for stirring up the thought process could have been given. 
- Which tools could have been the most useful in managing malaria affected children, easing up the burden from mothers? Elimination and prevention - mothers' perception of most effective tool? And that tool's accessibility and use is the next question. 

Does the article adequately reference differing views and opinions?

Partly

Are all factual statements correct, and are statements and arguments made adequately supported by citations?

Yes

Is the Open Letter written in accessible language?

Yes

Where applicable, are recommendations and next steps explained clearly for others to follow?

Yes

Is the rationale for the Open Letter provided in sufficient detail?

Yes

Reviewer Expertise:

I am a Public Health specialist with spl interest in neglected diseases and eliminable diseases. I take keen interest in Research which influences policies.

I confirm that I have read this submission and believe that I have an appropriate level of expertise to confirm that it is of an acceptable scientific standard.

# Peer review: Reviewer response for version 1

**DOI:** 10.21956/gatesopenres.14348.r29151

**Published:** 2020-08-10

**Authors:** Qi Zhang

**Affiliations:** 1 Boston University, Boston, MA, USA

Malaria has put stress on not only human health but also working productivity. The impact is particularly severe to vulnerable groups such as agricultural smallholders, as the study subjects in this research. This letter moves a step forward on reducing gender inequality using a case on the impacts of malaria on both male and female farmers’ agricultural productivities in sub-Saharan Africa.  The merit should be appreciated.  Meanwhile, there are several issues raising concern, which are discussed below:

  

- In the Abstract, it would be more precise to include the key word “agricultural productivity” somewhere or in the sentence addressing the research gap (quoted “little progress has been made in understanding how eliminating malaria could affect gender inequality within agricultural households”).
- In the Zambia case in Introduction, it appears more logical by deleting “lost”, being “a reduction in work days due to malaria morbidity”. Thus, high malaria morbidity may lead to reduced working days.
- The literature review is incomplete and unbalanced. It is suggested to more comprehensively and objectively to summarize current knowledge in the literature. For instance, it sounds over-stated by asserting “no such study have done so”. Although gender inequality specific to malaria and agricultural productivity might not be the main focus, previous studies have touched on the difference between male and female farmers. In Badiane and Ulimwengu (2013 ^1^), results include higher agricultural efficiency by female farmers than male counterparts, and a negative relationship between malaria incidence and the efficiency. It is not a requirement to cite this paper, but to urge a more accurate reflection of the literature. Please note this does not mean citing as many papers as one can, but it needs a better display of the full picture.
- For the definition of the household, which might be explained in detail in previous work, it needs clarification for the consistency. The 2010 census definition does not include the farm size, but farm size seems the only major characteristic that defines a household. Another unclear property relates to the farming size (less than 10 hectare). What would a unit with 10+ ha farming area be called?
- A question for “Women are often required to farm land that they do not own, such as land belonging to their husband or male relatives”: would farm plots belong separately to the female member and husband?  In many cases, a farm plot belongs to a household in which all members own and manage the farm plot. Please explain more. This is important given the later information of protective policies on women’s land rights.
- “Most estimates for caregiving days necessary per malaria case for households in sub-Saharan Africa ranged from one to six days.” What is the time scale of this range (e.g. 1-6 per week, per month, or per year)?
- “Further, we estimate that women provide care in 80% of cases involving younger children and 70% of cases involving older children.” If these two numbers are directly assumed, replace “estimate” to “assume”.
- Regarding “inequality” and “difference”, inequality is more conclusive while difference is more descriptive. When referring to statistics, it is suggested to use “difference”, such as in the description of caregiving days between male and female farmers in Fig. 1. The difference between their times spent on caregiving could be evidence reflecting gender inequality. If agreed, please check the terms throughout the paper.
- The research agenda is timely and necessary, which is grateful. Eradicating malaria is an ambitious and arduous process, which needs long-lasting research and practices. It is even more important to mitigate and eradicate gender inequality along the way to promote human health particularly for younger generations.

Does the article adequately reference differing views and opinions?

Partly

Are all factual statements correct, and are statements and arguments made adequately supported by citations?

Partly

Is the Open Letter written in accessible language?

Yes

Where applicable, are recommendations and next steps explained clearly for others to follow?

Yes

Is the rationale for the Open Letter provided in sufficient detail?

Yes

Reviewer Expertise:

human-environmental nexus; rural poverty; agricultural livelihoods

I confirm that I have read this submission and believe that I have an appropriate level of expertise to confirm that it is of an acceptable scientific standard, however I have significant reservations, as outlined above.

## References

[1] : Malaria incidence and agricultural efficiency in Uganda. *Agricultural Economics*.2013;44(1) : 10.1111/j.1574-0862.2012.00626.x 15-23 10.1111/j.1574-0862.2012.00626.x
